# SE-CPPA: A Secure and Efficient Conditional Privacy-Preserving Authentication Scheme in Vehicular Ad-Hoc Networks

**DOI:** 10.3390/s21248206

**Published:** 2021-12-08

**Authors:** Mahmood A. Al-Shareeda, Mohammed Anbar, Selvakumar Manickam, Iznan H. Hasbullah

**Affiliations:** National Advanced IPv6 Centre (NAv6), Universiti Sains Malaysia (USM), Penang 11800, Malaysia; m.alshareeda@nav6.usm.my (M.A.A.-S.); selva@usm.my (S.M.); iznan@usm.my (I.H.H.)

**Keywords:** Vehicular Ad-Hoc Networks (VANETs), identity-based cryptography, impersonation attacks, bilinear pair cryptography, privacy-preserving, side-channel attacks

## Abstract

Communications between nodes in Vehicular Ad-Hoc Networks (VANETs) are inherently vulnerable to security attacks, which may mean disruption to the system. Therefore, the security and privacy issues in VANETs are entitled to be the most important. To address these issues, the existing Conditional Privacy-Preserving Authentication (CPPA) schemes based on either public key infrastructure, group signature, or identity have been proposed. However, an attacker could impersonate an authenticated node in these schemes for broadcasting fake messages. Besides, none of these schemes have satisfactorily addressed the performance efficiency related to signing and verifying safety traffic-related messages. For resisting impersonation attacks and achieving better performance efficiency, a Secure and Efficient Conditional Privacy-Preserving Authentication (SE-CPPA) scheme is proposed in this paper. The proposed SE-CPPA scheme is based on the cryptographic hash function and bilinear pair cryptography for the signing and verifying of messages. Through security analysis and comparison, the proposed SE-CPPA scheme can accomplish security goals in terms of formal and informal analysis. More precisely, to resist impersonation attacks, the true identity of the vehicle stored in the tamper-proof device (TPD) is frequently updated, having a short period of validity. Since the MapToPoint hash function and a large number of cryptography operations are not employed, simulation results show that the proposed SE-CPPA scheme outperforms the existing schemes in terms of computation and communication costs. Finally, the proposed SE-CPPA scheme reduces the computation costs of signing the message and verifying the message by 99.95% and 35.93%, respectively. Meanwhile, the proposed SE-CPPA scheme reduces the communication costs of the message size by 27.3%.

## 1. Introduction

Annually, approximately 1.3 million persons die, and between 20 and 50 million more persons are non-fatally injured as a result of a road traffic accidents [1,2]. Therefore, the technology of Vehicular Ad-Hoc Networks (VANETs) is expected to play a major role in reducing the number of accidents and increasing road safety [3,4]. VANETs have attracted increasing attention from academia, the motor industry, and even the government in recent years [5].

**Figure 1:**
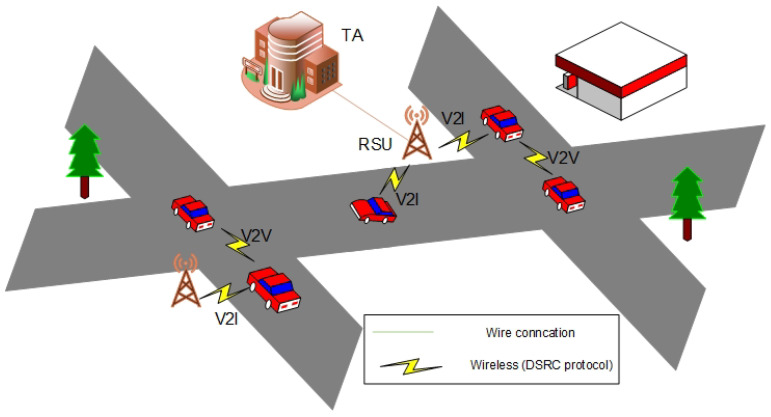
The main structure of the VANET.

VANETs are an extreme case of Mobile Ad-Hoc Networks (MANETs), in which the vehicle nodes are highly mobile. The main structure includes three components of the VANET, namely a trusted authority (TA), some fixed road-side units (RSUs), and many mobility on-board units (OBUs), as shown in Figure 1. Each vehicle has OBU to share safety traffic-related messages with others or neighbor RSU via vehicle-to-vehicle (V2V) communication and vehicle-to-infrastructure (V2I) communication, respectively. More precisely, the main goals of intelligent transport system (ITS) are to offer safety improving, and driving efficiency in the road environment. With these goals in mind, VANETs have become a promising technology.

Nevertheless, this advantage comes with issues of security, privacy, and performance efficiency. Hence, these issues should be carefully considered in VANETs [6,7,8]. The security issue is crucial in V2V and V2I communications. Due to the inherently insecure nature of the communication between nodes, a VANET is vulnerable to security attacks which may mean disruption to the system [9,10]. It is possible for attackers to replay, modify, and intercept legitimate transmitted safety traffic-related messages. Furthermore, by using a side-channel attack [11,12,13,14], the attacker could obtain the true identity of a vehicle stored in the tamper-proof device (TPD). Consequently, this attacker is being considered as impersonates registered vehicles in VANETs. Once the impersonation attacks broadcast fake messages, it results in creating road chaos and traffic incidents, or even inducing wrong decisions by other vehicles [15,16,17,18,19,20,21].

In addition, the privacy issue is also critical. In a VANET, attackers might obtain the vehicle’s true identity and trace its journey by investigating the captured messages. Such an attack exposes the driver’s personal and other vehicular details, and it can be leveraged to carry out other forms of attacks. Thus, the drivers would be reluctant to use the VANET technology.

Apart from the requirements of security and privacy, performance efficiency is also important in V2V and V2I communications. Within 100–300 ms, the vehicle must send exchanged information according to the DSRC technology. For instance, based on the communication range of vehicle or RSU, when there are 100 vehicles, the receiver is required to authenticate 333–1000 messages per second. Each message can certainly be signed and tested in a secure communication.

Therefore, the received messages should verify the authenticity and validate the integrity by receivers (RSUs or OBUs) before accepting them. Anonymous communication is needed to preserve privacy and to fulfill the unlinkability requirement for the drivers. The existing Conditional Privacy-Preserving Authentication (CPPA), based on either public key infrastructure, group signature, or identity, can be used to satisfy both security and privacy in VANETs. Nevertheless, these schemes have several drawbacks, as discussed in Section 2.

This paper proposes a Secure and Efficient Conditional Privacy-Preserving Authentication (SE-CPPA) scheme for VANETs in order to address drawbacks in the existing CPPA schemes. More precisely, the main contributions of the proposed SE-CPPA scheme are as follows:

- First, this efficient bilinear pair cryptography based on the conditional privacy-preserving authentication (SE-CPPA) scheme satisfies the security and privacy requirements.
- Second, since the vehicle’s true identity is regularly updated at short intervals of time, the proposed SE-CPPA scheme is resistant to impersonation attacks, as attackers are unable to launch side-channel attacks for obtaining the vehicle’s true identity.
- Third, since the signing and verifying of the messages do not employ a MapToPoint hash operation function, the proposed SE-CPPA scheme has a lower overhead compared to the existing schemes based on bilinear pair cryptography.

The remainder of this paper is structured as follows. The existing CPPA schemes for VANETs are reviewed in Section 2. Section 3 introduces the background for the proposed SE-CPPA scheme. The phases of the proposed SE-CPPA schemes are presented in detail in Section 4. Section 5 introduces a security analysis and comparison in this paper. In Section 6, the performance efficiencies of the SE-CPPA and the existing CPPA schemes are evaluated and compared. Lastly, our conclusion is introduced in Section 7.

## 2. Related Work

In this section, the existing CPPA schemes for VANETs are briefly reviewed. The following categories for the existing CPPA schemes are, namely: Public key infrastructure, group signature, and Identity. These categories will be separately reviewed in the next subsections.

### 2.1. Public Key Infrastructure-Based CPPA

The main idea of the public key infrastructure-based CPPA schemes [22,23,24,25,26,27,28,29,30] is to preload a massive pool of private/public keys and their matching certificates to the OBUs of vehicles, generated by the TA during the registration process. This approach supports privacy-preserving, since a massive pool of private/public keys and their matching certificates are preloaded in advance.

Joshi et al. [29] designed an event-triggered authentication scheme that sends messages to investigate problems regarding security in the VANET. Asghar et al. [30] designed a feasible PKI-CPPA scheme to tackle the process of authenticating requests, in which the size of the Certificate Revocation List (CRL) is linear. Thus, this scheme enhances the scalability of vehicles’ obtaining services.

Nevertheless, the main limitations of a public key infrastructure based-CPPA schemes are: (i) preloading a massive pool of private/public keys and their matching certificates to the OBUs of the vehicles makes the management of the certificates a serious burden; (ii) the storage of a vehicle in a VANET is limited, since massive keys and their matching certificates are preloaded; (iii) there are additional computational and communication costs, since the certificate is included in the message signature, and the verifier must verify these certificates as well.

### 2.2. Group Signature Based-CPPA

To address the limitations regarding a public key infrastructure based-CPPA scheme, several researchers design a group signature based-CPPA scheme [31,32,33,34]. These schemes enable the members of the group to sign on behalf of the whole group anonymously. In the event of a dispute, the group manager could retrieve the identification of the sender. Thus, the existing group signature-based CPPA schemes preserve the anonymity of secured authenticated messages. Besides, these schemes ensure secure communication with conditional privacy. Therefore, signing the messages with these schemes can hide the signer’s identity.

Nevertheless, the main limitations of a group signature based-CPPA schemes are: (i) the whole group must be reconstructed; (ii) it is not easy for nodes’ VANETs to update their private keys; (iii) the adversary identifies the group members when the size of the group is small; and (iv) once the number of vehicles revoked is high, the signature’s verification technique becomes time-consumed for VANETs.

### 2.3. Identity-Based CPPA

To address the limitations regarding a public key infrastructure-based CPPA and group signature-based CPPA schemes, several researchers propose an identity-based CPPA scheme [35,36,37,38,39,40,41]. The primary insight of identity based-CPPA scheme is to extract the public key from the identity information, while the TA creates a private key with the same information. The sender signs the message using its private key, and the verifier can verify this signature by using the sender’s public key.

Bayat et al. [36] designed an identity-based CPPA scheme to save the TA’s private key on the TPD of the OBU on the vehicle. However, the revocation requirement is not satisfied in the scheme designed by [36], which is vulnerable to impersonation attacks. Lei Zhang et al. [37] designed a distributed aggregate CPPA scheme by using a realistic TPD rather than an ideal TPD, since this is more practical. Bayat et al. [38] designed an identity-based CPPA to propose an RSU-based authentication scheme that uses bilinear pair operations to secure the communications. Pournaghi et al. [39] designed an identity-based CPPA to provide secure communications between nodes for VANETs. Nevertheless, it is vulnerable to replay attacks. Zhong et al. [40] found that the CPPA process of the scheme proposed by Lei Zhang et al. [37] introduced a massive computational cost, and it did not indicate who is the aggregator in the aggregation process. Bayat et al. [41] introduced an identity based-CPPA scheme without using an online RSU, for the sake of the security of the communication in the VANET.

Nevertheless, the two evident limitations of an identity based-CPPA scheme are: (i) the vehicle’s true identity preloaded by the TA is vulnerable to impersonation attacks by launching side-channel attacks, since it is not updated rapidly enough; and (ii) the MapToPoint hash function and a large number of cryptography operations are used, which cause a huge performance overhead by the verifier. To address these issues, a Secure and Efficient Conditional Privacy-Preserving Authentication (SE-CPPA) scheme is proposed for resisting impersonation attacks and achieving better performance efficiency during the broadcasting process. The proposed SE-CPPA scheme regularly updates the vehicle’s true identity for the short period of validity assigned by the TA. As well, it does not use the MapToPoint hash function and a large number of cryptography operations.

## 3. Preliminaries

This section first presents the network model of the proposed scheme; this is followed by a presentation of the security and privacy requirements for VANETs, and finally, the bilinear pair cryptography (BPC) used in the proposed SE-CPPA scheme is defined.

### 3.1. Network Model

As shown in Figure 1, the main structure of the network model for the proposed SE-CPPA scheme includes three components: TA, RSU, and OBU.

- TA: TA is a fully trusted unit with a great number of resources in terms of computation and communication costs. The TA issues the public parameters of the system for each node in VANETs, and transmits them to each respective node.
- RSU: An RSU is a wireless base station deployed on the road as a bridge interface between the TA and the OBUs. Since RSU has a TPD to save a sensitive information, RSU is considered as a trusted entity in this paper. An RSU connects with the TA by wired technology and connects with vehicles by wireless technology.
- OBU: Each vehicle has an OBU to allow the vehicle to process, receive, and broadcast messages. Each OBU has a TPD that is usually used to keep secrets.

### 3.2. Security and Privacy Requirements

To maintain the security and privacy of V2V and V2I communications in VANETs, the proposed SE-CPPA scheme should fulfill the following requirements.

- Authentication and integrity: The vehicle or RSU must be able to identify any alteration of the received message, by checking the authentication process and validating integrity, in order to ensure the security of the communications in the VANET.
- Identity privacy-preserving: An attacker must not be able to retrieve the true identity of the vehicle by the capturing messages transmitted. Therefore, the vehicle’s true identity must be kept anonymous from the other legal and illegal nodes for the sake of ensuring the privacy of the drivers.
- Traceability and revocation: The TA must be able to retrieve the true identity of the vehicle from its message in the event of a dispute, so as to avoid misbehaving vehicles from denying their responsibility for a disruption of the system by broadcasting false messages to other registered vehicles.
- Unlinkability: An attacker must not be able to cross-match several messages transmitted by the same source for ensuring privacy-preserving.
- Resistance to security attack: A secure proposed SE-CPPA scheme should resist the following security attacks.
  – Replay attacks.
    The malicious nodes aim to replay a previously generated legitimate signature to the recipient.
  – Modification attacks.
    The malicious nodes aim to alter the authentic message and broadcast that to other users.
  – Impersonation attacks.
    After launching a side-channel attack to retrieve the true identity of the vehicle, the malicious nodes aim to impersonate an authenticated node to broadcast a legitimate message to other nodes. Therefore, the vehicle’s true identity must be frequently updated within a short period of validity.
  – Man-In-The-Middle attacks.
    The malicious nodes aim to intercept two sides of the communication and perform data tampering and sniffing.

### 3.3. Bilinear Pair Cryptography (BPC)

Let $G_{1}$ and $G_{2}$ be a cyclic additive and a cyclic multiplicative group, respectively. Both $G_{1}$ and $G_{2}$ have the same generator *P* and prime order *p*.

BPC is a map *e*: $G_{1}*G_{1}$→$G_{2}$ which has the following properties.

- **Bilinearity:** Every *X*, *Y*∈$G_{1}$ and *a*, *b*∈$Z_{p}^{*}$, $e\left(aX,bY\right)={\left(X,Y\right)}^{ab}$.
- **Non-degeneracy:***e*:(P,P)≠ 1.
- **Computability:** Every $X,Y\in G_{1}$, there is an efficient approach to calculate e(X,Y).

## 4. Proposed Scheme

In this section, the proposed SE-CPPA scheme is discussed. More precisely, the proposed SE-CPPA scheme consists of seven phases, namely initialization, vehicle registration, mutual authentication, message signing, individual-signature verification, batch-signature verification, and updating the vehicle’s true identity. Table 1 presents the notation used, and their description in the following phases.

We noted that an external attacker has the ability to impersonate legitimate vehicles by launching side channel attack to disclose the sensitive information stored on TPD of legitimate vehicle when information is not updated; in the result, the external attacker should be possible to forge a secret.

### 4.1. Initialization

As explained in Section 3.3, the TA executes the initial public parameters of the BPC for the system in the following steps:

- Consider $G_{1}$ and $G_{2}$ be groups of a cyclic additive a cyclic multiplicative, respectively, with the same prime order *q* and generator *P*. Consider *e*: $G_{1}\cdot G_{1}$∗$G_{2}$ as a bilinear pairing.
- The TA chooses three functions of secure cryptographic hash $h_{1}:G\to Z_{q}^{*}$, $h_{2}:{\left\{0,1\right\}}^{*}\times {\left\{0,1\right\}}^{*}\times G\to Z_{q}^{*}$, and $h_{3}:{\left\{0,1\right\}}^{*}\to Z_{q}^{*}$.
- The TA chooses a random integer $s_{TA}\in Z_{q}^{*}$ to be the TA’s master private key, and then calculates $P_{TA}$= $s_{TA}P$ to be its matching master public key.
- The TA preloads the system’s public parameters {$G_{1}$, $G_{2}$, *P*, *q*, $P_{TA}$, $h_{1}$, $h_{2}$, $h_{3}$} and the TA master private key $s_{TA}$ in each TPD on RSU.

### 4.2. Vehicle Registration

**Figure 2:**
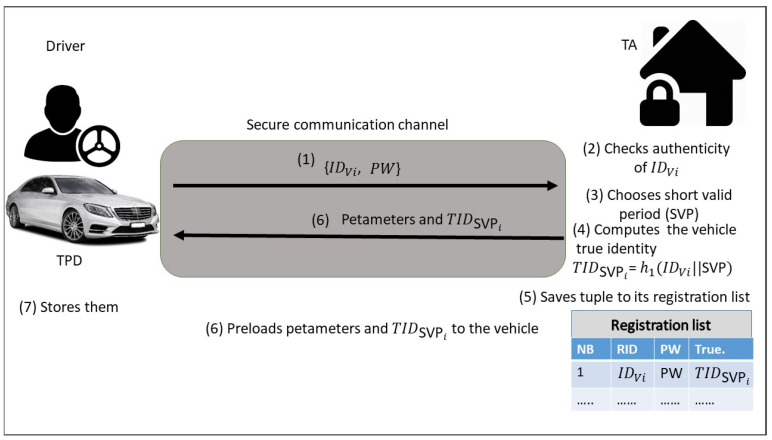
Process of vehicle registration phase.

Prior to the vehicle leaving the factory, the vehicle registration phase via a secure channel (offline) should be executed. Due to the core problem study in this paper, the vehicle’s true identity should be regularly updated to avoid side channel attack. Hence, the proposed scheme is resisting impersonation attacks. As shown in Figure 2, the TA registers each vehicle as follows:

- The driver of the vehicle submits the personal information including the identity $ID_{vi}$ and password Pwd to the TA via a secure communication network.
- After the personal information is received, the TA first starts the authenticity of $ID_{vi}$.
- After the TA chooses a short period of validity SVP, the TA computes the vehicle’s true identity $TID_{SVP_{i}}=h_{1}(ID_{vi}\left||SVP\right)$.
- The TA saves the tuple {$ID_{vi}$, Pwd, $TID_{SVP_{i}},SVP$} to its vehicle registration list, and then preloads the system’s public parameters {$G_{1}$, $G_{2}$, *P*, *q*, $P_{TA}$, $h_{1}$, $h_{2}$, $h_{3}$} and $TID_{SVP_{i}}$ into TPD of $OBU_{i}$ on the vehicle.

### 4.3. Mutual Authentication

Before the vehicle signs and verifies exchanged messages, it should be authorized with a nearby RSU. Therefore, when a vehicle enters the communication area of an RSU, it starts to broadcast an entering request message. After the messages are validated, the RSU sends a signature key $SK_{svt}$ to the vehicle with a chosen timestamp svt that will be valid for a short period of time. To execute the mutual authentication process, the following process should be done.

- The vehicle randomly selects a value $\zeta_{i}$∈$Z_{q}^{*}$ and then calculates the following pseudonym ID:
  (1) $$\begin{array}{cc} pid_{i} & =\{pid_{i}^{1},pid_{i}^{2}\}\\ \; & =\{\zeta_{i}P,TID_{SVP_{i}}⊕h_{1}\left(\zeta_{i}P_{TA}\right)\} \end{array}$$
- The vehicle broadcasts the join request {$pid_{i}^{1},pid_{i}^{2},\delta_{RJ}$} to a nearby RSU, where $\delta_{RJ}=h_{2}(TID_{SVP_{i}}||pid_{i}^{1}||pid_{i}^{2})$.
- The RSU obtains the vehicle’s true identity using the following equation,
  (2) $$\begin{array}{c} TID_{SVP_{i}}=pid_{i}^{2}⊕h_{1}\left(s_{TA}\cdot pid_{i}^{1}\right) \end{array}$$
- The RSU then computes the validity of the request to join {$pid_{i}^{1},pid_{i}^{2},\delta_{RJ}$} by calculating
  (3) $$\begin{array}{c} \delta_{RJ}\overset{?}{=}h_{2}(TID_{SVP_{i}}||pid_{i}^{1}||pid_{i}^{2}). \end{array}$$
- The RSU then checks the vehicle’s true identity on its certificate revocation list (CRL). If it is on the list, the request is rejected by the RSU for joining the session. Otherwise, the RSU continues the process.
- The RSU computes the signature key $SK_{svt}$ of the vehicle’s true identity from the request to join, as follows:
  (4) $$\begin{array}{c} SK_{svt}=s_{TA}\cdot h_{3}(pid_{i}^{1}||pid_{i}^{2}||svt). \end{array}$$
  Here, svt is the expiry of a certain brief period of validity of the timestamp of the created signature key.
- The RSU sends the message of the acceptance of the joining {$SK_{svt}^{ENC},pid_{i}^{1},pid_{i}^{2}\delta_{AJ}$} to the vehicle, where $SK_{svt}^{ENC}=SK_{svt}⊕h_{2}\left(s_{TA}\cdot pid_{i}^{1}\right)$ and $\delta_{AJ}=h_{2}(SK_{svt}||pid_{i}^{1}||pid_{i}^{2})$.
- The vehicle retrieves the signature key from the message of acceptance {$SK_{svt}^{ENC},svt,pid_{i}^{1},pid_{i}^{2}\delta_{AJ}$} by calculating $SK_{svt}=SK_{svt}^{ENC}⊕h_{2}\left(\zeta_{i}P_{TA}\right)$, and then verifies the validity of the acceptance by utilizing the following equation.
  (5) $$\begin{array}{c} \delta_{AJ}\overset{?}{=}h_{2}(SK_{svt}||pid_{i}^{1}||pid_{i}^{2}||svt). \end{array}$$

The process in the proposed SE-CPPA scheme of preloading, as introduced in [42,43], fulfills the requirements of security and privacy of $\zeta_{l}$, the pseudonym IDs, and the signature keys. The TA preloads a new list of $\zeta_{l}$, pseudonym IDs, and signature keys, used for an svt for each vehicle moving in a VANET; close to the expiration time, they are renewed with a new pseudonym ID and pool of signature keys.

### 4.4. Message Signing

After the signature key, $SK_{svt}$ of the vehicle’s true identity has been received, the vehicle is taken into consideration as an authorized component in the VANET. The vehicle signs and sends safety traffic-related messages $m_{i}$ to other vehicles and RSUs in the VANET. This is executed in the phases listed below.

- The vehicle computes the message signature $\delta_{m_{i}}=SK_{svt}\cdot h_{3}(m_{i}\left||ts\right)$, where ts is a current timestamp.
- The vehicle then broadcasts the signature tuple {$pid_{i}^{1}$, $pid_{i}^{2}$, $m_{i}$, svt, ts, $\delta_{m_{i}}$} to the neighboring recipient.

### 4.5. Individual Signature Verification

At a given point of time, the main aim of this method is to verify only one message with the signature $\delta_{m_{i}}$ on the message $m_{i}$ by the recipient (OBU or RSU). Once having received the signed message $m_{i}$, and before accepting it, the recipient checks the authenticity of the node and the validity of the message. This guarantees that no illegitimate recipient is impersonating a legitimate recipient or sending fake messages. The recipient receives an authentic signature $\delta_{m_{i}}=SK_{svt}\cdot h_{3}(m_{i}\left||ts\right)$ on the message $m_{i}$ from the vehicle with a pseudonym ID $pid_{i}$ and timestamp ts, where i=1, and checks its authenticity and validity following the steps below.

- Once the signature tuple {$pid_{i}^{1}$, $pid_{i}^{2}$, $m_{i}$, ts, $\delta_{m_{i}}$} has been received, the vehicle first verifies the timestamp TS and svt validity. If (ts > $ts_{r}-ts_{▽}$), where $ts_{r}$ is the time of receiving and $ts_{▽}$ is a predefined delay, then ts is considered as fresh. Otherwise, the message is rejected.
- The vehicle uses the public parameters and functions of the system and signature $\delta_{m_{i}}=SK_{svt}\cdot h_{3}(m_{i}\left||ts\right)$ on the message $m_{i}$. When the following Equation (6) holds, the vehicle accepts it.

(6) $$e:\left(\delta_{m_{i}}P\right)=e:(h_{2}(pid_{i}^{1}||pid_{i}^{2}\left||svt\right)h_{3}(m_{i}||ts),P_{TA})$$

### 4.6. Batch-Signature Verification

The main aim of this method is to authenticate a batch of signature messages $\delta_{m_{i}}=\left\{\delta_{m_{1}},\delta_{m_{2}},\delta_{m_{3}},\ldots ,\delta_{m_{n}}\right\}$ on *n* traffic-related messages $m_{i}=\left\{m_{1},m_{2},m_{3},\ldots ,m_{n}\right\}$ from *n* vehicles with *n* pseudonym IDs $pid_{i}=\{pid_{1},pid_{2}$, $pid_{3},\ldots ,$ $pid_{n}\}$. The verifying recipient checks its authenticity and validity, as shown in the following steps.

- The vehicle verifies the validity of ts and svt. If (ts > $ts_{r}-ts_{▽}$), ts is considered as fresh. Otherwise, the message is rejected.
- The vehicle uses the small exponent technique [44,45] to avoid denying the validity of the message sent in the SE-CPPA proposed. The vehicle generates a random vector $\gamma_{i}$ = {$\gamma_{1},\gamma_{2},\gamma_{3},\ldots ,\gamma_{n}$}, where $\gamma_{i}\in$ $\left[1:2^{t}\right]$ and *t* is a small value.
- To accept them, the vehicle checks whether

(7) $$e\left(\sum_{i=1}^{n}\left(\gamma_{i}\cdot \delta_{m_{i}}\right)\right)\cdot P=e\left(\sum_{i=1}^{n}\gamma_{i}\cdot h_{2}(pid_{i}^{1}||pid_{i}^{2}\left||svt\right)h_{3}(m_{i}||ts),P_{TA}\right)$$

### 4.7. Updating the Vehicle’s True Identity

**Figure 3:**
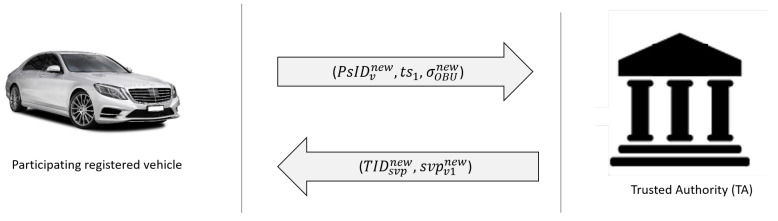
Update vehicle true identity process.

In order to resist impersonation attacks, the vehicle’s true identity stored in the TPD should be frequently updated through an online process and annual examination. However, if one were to wait for the next annual examination to update the vehicle’s stored true identity, the adversary would have a long enough period to retrieve a vehicle’s true identity, something that can disrupt the entire VANET by impersonating as an authorized vehicle. During the vehicle, true identity SVP is close to expired; the registered vehicle could not have requested update the lists before the process of $TID_{svp}$ is totally completed to avoid contradictions. As presented in Figure 3, the following steps are used to update the vehicle’s true identity saved in the vehicle by using an online process:

- The vehicle selects a random value $k\in Z_{q}^{*}$ and calculates $PsID_{i,1}=kP$ and $PsID_{i,2}=TID_{svp}⊕h_{1}\left(k\cdot P_{TA}\right)$. Then, the vehicle broadcasts an update message {$PsID_{v,new}$, $ts_{1}$, $\delta_{OBU_{new}^{i}}$} to the TA, where $PsID_{v}^{new}$ = {$PsID_{i,1}=kP$, $PsID_{i,2}=TID_{svp}⊕h_{1}\left(k\cdot P_{TA}\right)$} and $\delta_{OBU_{new}^{i}}$ = $h_{3}(TID_{svp}∥PsID_{i,1}∥PsID_{i,2}∥$ $ts_{1})$.
- Once the TA receives the update message {$PsID_{v,new}$, $ts_{1}$, $\delta_{OBU_{new}^{i}}$}, the timestamp $ts_{1}$ validity is tested. If $ts_{1}$ is freshness, then the TA computes the vehicle’s old true identity of the authenticated vehicle $TID_{svp}=PsID_{i,2}⊕h_{1}\left(k\cdot P_{TA}\right)$. The TA tests whether $\delta_{OBU_{new}^{i}}$ $\overset{?}{=}$ $h_{3}(TID_{svp}∥PsID_{i,1}∥PsID_{i,2}∥$ $ts_{1})$ holds. The TA then checks whether the tuple ($TID_{svp},svp,ID_{vi}$) existing in its registration list; else TA checks the svp validity.
- When the svp has expired, a new short period of validity $svp^{New}$ is chosen by the TA. Then, the TA generates a new true identity $TID_{svp}^{New}=h_{3}\left(ID_{vi}∥svp^{New}\right)$ for the vehicle. It will be discarded if svp is still valid.
- The TA sends an accepted update message ($TID_{svp}^{\text{New-enc}},$ $svp^{New}$) to the vehicle, where $TID_{svp}^{\text{New-enc}}$ = $ID_{svp}^{New}⊕h_{1}\left(s_{TA}\cdot PsID_{i,1}\right)$.
- Finally, the vehicle retrieves its new true identity $TID_{svp}^{New}$ = $TID_{svp}^{\text{New-enc}}⊕h_{1}\left(s_{TA}\cdot PsID_{i,1}\right)$ to get the new true identity of the vehicle.

## 5. Security Analysis and Comparison

This section presents the formal and informal analysis of the proposed SE-CPPA scheme. In addition, the security-based privacy requirements are listed.

### 5.1. Formal Analysis

The formal analysis presents the security proof regarding the verification equations; this is followed by a description of the steps of the random oracle model.

#### 5.1.1. Security Proof

**Theorem** **1.** *The equations utilized in the proposed SE-CPPA scheme are true.*

**Proof of Equation** **(6).** In individual-signature verification, the verifier checks the message using the following Equation (6).

$$\begin{array}{cc} \; & L\cdot H\cdot Se\left(\delta m_{i}\cdot P\right)\\ \; & =e\left(SK_{svt}h_{3}(m_{i}||ts),P\right)\\ \; & =e\left(s_{TA}h_{2}(pid_{i}^{1}||pid_{i}^{2}\left||svt\right)h_{3}(m_{i}||ts),P\right)\\ \; & =e\left(h_{2}(pid_{i}^{1}||pid_{i}^{2}\left||svt\right)h_{3}(m_{i}||ts),s_{TA}P\right)\\ \; & =e\left(h_{2}(pid_{i}^{1}||pid_{i}^{2}\left||svt\right)h_{3}(m_{i}||ts),P_{TA}\right)\\ \; & =R\cdot H\cdot S \end{array}$$

Hence, the individual signature verification correctness is true. □

**Proof of Equation** **(7).** In batch-signature verification, the verifier checks a large number of messages by using the following Equation (7). Proof of the correctness:

$$\begin{array}{cc} \; & L\cdot H\cdot Se\left(\sum_{i=1}^{n}\gamma_{i}\cdot \delta m_{i}\cdot P\right)\\ \; & =e\left(\sum_{i=1}^{n}\gamma_{i}\cdot SK_{svt}h_{3}(m_{i}||ts),P\right)\\ \; & =e\left(\sum_{i=1}^{n}\gamma_{i}\cdot s_{TA}h_{2}(pid_{i}^{1}||pid_{i}^{2}\left||svt\right)h_{3}(m_{i}||ts),P\right)\\ \; & =e\left(\sum_{i=1}^{n}\gamma_{i}\cdot h_{2}(pid_{i}^{1}||pid_{i}^{2}\left||svt\right)h_{3}(m_{i}||ts),s_{TA}P\right)\\ \; & =e\left(\sum_{i=1}^{n}\gamma_{i}\cdot h_{2}(pid_{i}^{1}||pid_{i}^{2}\left||svt\right)h_{3}(m_{i}||ts),P_{TA}\right)\\ \; & =R\cdot H\cdot S \end{array}$$

Hence, the batch-signature verification correctness is true. □

#### 5.1.2. Random Oracle Model

In order to analyze the security proof in the SE-CPPA scheme, the random oracle model analysis defines a game between an attacker ER and the challenger Ch. Once ER wins the game, it is easily retrieved from a valid faked signature. Furthermore, the proposed SE-CPPA scheme is secure for VANETs when ER is negligible for any attack.

**Theorem** **2.** *The proposed SE-CPPA scheme for VANETs is unforgeable against an adaptively chosen message attack under the random oracle model.*

**Proof.**  Assuming ER could forge a valid message of the signature tuple {$pid_{i}^{1}$, $pid_{i}^{2}$, $m_{i}$, svt, ts, $\delta_{m_{i}}$} for the message $m_{i}$, it would follow that a challenger Ch can be generated to resolve the ECDL problem with non-negligible probability by launching ER as a subroutine. □

Setup initialization phase: Challenger Ch first randomly chooses a value $s_{TA}$∈$Z_{q}^{*}$ as the system’s private key and computes $P_{TA}$ = $s_{TA}P$ as the system’s public key. Then, Ch broadcasts the public parameters and functions of the system to ER.

$Oracle-h_{1}$. Ch starts the $h_{list_{1}}$ with $\left(\alpha ,\tau h_{1}\right)$ form. After, ER receives a message with (α) form, Ch sees whether (α) is in $h_{list_{1}}$; if so, Ch transmits $\left(\tau h_{1}=h\left(\alpha \right)\right)$ to ER. Otherwise, Ch chooses $\tau h_{1}\in Z_{q}^{*}$ randomly and adds $\left(\alpha ,\tau h_{1}\right)$ into $h_{list_{1}}$. Then, ER broadcasts $\tau h_{1}=h\left(\alpha \right)$ to Ch.

$Oracle-h_{2}$. Ch starts the $h_{list_{2}}$ with $\left(pid_{i}^{1},pid_{i}^{2},\tau h_{2}\right)$ form. After, ER receives a message with $\left(pid_{i}^{1},pid_{i}^{2}\right)$ form, Ch tests whether $\left(pid_{i}^{1},pid_{i}^{2}\right)$ is in $h_{list_{2}}$; if so, Ch broadcasts $\tau h_{2}=h(pid_{i}^{1}||pid_{i}^{2}||\tau h_{2})$ to ER. Otherwise, Ch randomly chooses $\tau h_{2}\in Z_{q}^{*}$ and puts $\left(pid_{i}^{1},pid_{i}^{2},\tau h_{2}\right)$ into $h_{list_{2}}$. Then, ER broadcasts $\tau h_{2}=h(pid_{i}^{1}||pid_{i}^{2}||\tau h_{2})$ to Ch.

$Oracle-h_{3}$. Ch starts the $h_{list_{3}}$ with $\left(m_{i},ts,svt,\tau h_{3}\right)$ form. After ER receives a message with $\left(m_{i},ts,svt\right)$ form, Ch tests whether $\left(m_{i},ts,svt\right)$ is in $h_{list_{3}}$; if so, Ch broadcasts $\tau h_{3}=h(m_{i}||ts||svt||\tau h_{3})$ to ER. Otherwise, Ch chooses $\tau h_{3}\in Z_{q}^{*}$ randomly and puts $\left(m_{i},ts,svt,\tau h_{3}\right)$ into $h_{list_{3}}$. Then, ER broadcasts $\tau h_{3}=h(m_{i}||ts||svt||\tau h_{3})$ to Ch.

Sign Oracle: Once ER sends a sign request, Ch calculates three random numbers, $h_{i,2}$; $h_{i,3}$; $\sigma_{m,i}\in Z_{q}^{*}$, and a random point $pid_{i}^{2}$∈*G*. Then, Ch computes $P_{TA}$ = ($\sigma_{m,i}P/h_{i,2}\cdot h_{i,3}$). Ch puts $\left(pid_{i}^{1},pid_{i}^{2},\tau h_{2}\right)$ into $h_{list_{2}}$ and $\left(m_{i},ts,svt\right)$ into $h_{list_{3}}$. Finally, Ch generates the message of the signature tuple {$pid_{i}^{1}$, $pid_{i}^{2}$, $m_{i}$, svt, ts, $\delta_{m_{i}}$} and transmits it to ER. The reply is a valid sign-oracle, since the message of the signature tuple {$pid_{i}^{1}$, $pid_{i}^{2}$, $m_{i}$, svt, ts, $\delta_{m_{i}}$} fulfills the following Equation:

$$\begin{array}{cc} \sigma_{m_{i}}\cdot P & =h_{i,2}\cdot h_{i,3}P_{TA}\\ \sigma_{m_{i}}\cdot P & =h_{i,2}h_{i,3}\cdot \left(\sigma_{m,i}P/h_{i,2}\cdot h_{i,3}\right)\\ \sigma_{m_{i}}\cdot P & =\left(h_{i,2}h_{i,3}/h_{i,2}\cdot h_{i,3}\right)\sigma_{m,i}P\\ \; & =\sigma_{m,i}P \end{array}$$

Output: Finally, ER outputs the message of the signature tuple {$pid_{i}^{1}$, $pid_{i}^{2}$, $m_{i}$, svt, ts, $\delta_{m_{i}}$}. Ch tests the message using the following Equation (8):

(8) $$\sigma_{m_{i}}P=h_{i,2}\cdot h_{i,3}P_{TA}$$

Once (8) does not hold, the game is finished by Ch.

According to the Cross Lemma, ER can output another message of signature tuple {$pid_{i}^{1}$, $pid_{i}^{2}$, $m_{i}$, svt, ts, $\delta_{m_{i}}$} that achieves the following Equation (9):

(9) $$\sigma_{m_{i}}^{*}P=h_{i,2}^{*}\cdot h_{i,3}^{*}P_{TA}$$

From Equations (8) and (9), it can be obtained

$$\begin{array}{cc} (\sigma_{m_{i}}-\sigma_{m_{i}}^{*})P & =\sigma_{m_{i}}P-\sigma_{m_{i}}^{*}P\\ \; & =\left(h_{i,2}\cdot h_{i,3}P_{TA}\right)-\left(h_{i,2}^{*}\cdot h_{i,3}^{*}P_{TA}\right)\\ & =\left(h_{i,2}\cdot h_{i,3}\right)-\left(h_{i,2}^{*}\cdot h_{i,3}^{*}\right)P_{TA}\\ \; & =\left(h_{i,2}\cdot h_{i,3}\right)-\left(h_{i,2}^{*}\cdot h_{i,3}^{*}\right)s_{TA}\cdot P \end{array}$$

Then, we can get $\left(\sigma_{m_{i}}-\sigma_{m_{i}}^{*}\right)=\left(h_{i,2}\cdot h_{i,3}-h_{i,2}^{*}\cdot h_{i,3}^{*}\right)$ $s_{TA}$ mod P. Ch resolves the ECDL problem by calculating $\left(\sigma_{m_{i}}-\sigma_{m_{i}}^{*}\right).{\left(h_{i,2}\cdot h_{i,3}-h_{i,2}^{*}\cdot h_{i,3}^{*}\right)}^{-1}$. However, since the difficulty of the ECDL problem with non-negligible probability, the proposed SE-CPPA scheme for VANETs is unforgeable against an adaptively chosen message attack under the random oracle model.

### 5.2. Informal Analysis

In this subsection, the proposed SE-CPPA scheme is shown below to fulfill the following security and privacy requirements for VANETs.

- Message integrity and authentication:
  Consistent with Theorem 2, when the problem of ECDLP is hard to solve, then no attacker can generate a legal message of the signature tuple {$pid_{i}^{1}$, $pid_{i}^{2}$, $m_{i}$, svt, ts, $\delta_{m_{i}}$} in a specified polynomial time. Thus, the message of the signature tuple fulfills the equation *e*:$\left(\delta_{m_{i}}P\right)$ = *e*:$(h_{2}(pid_{i}^{1}$ $||pid_{i}^{2}\left||svt\right)$ $h_{3}(m_{i}||ts),P_{TA})$, and so the proposed EPBC-CPPA can ensure message integrity and authentication.
- Identity privacy-preserving:
  Assume that an authorized vehicle sends a message of signature tuple {$pid_{i}^{1}$, $pid_{i}^{2}$, $m_{i}$, svt, ts, $\delta_{m_{i}}$} to neighbouring RSUs or vehicles in a VANET, where $pid_{i}=\left\{pid_{i}^{1},pid_{i}^{2}\right\}$ = $\left\{\zeta_{i}P,TID_{SVP_{i}}⊕h_{1}\left(\zeta_{i}P_{TA}\right)\right\}$ and $\zeta_{i}$∈$Z_{q}^{*}$. In order to obtain the vehicle’s true identity, the attacker should calculate $TID_{SVP_{i}}=pid_{i}^{2}⊕h_{1}\left(s_{TA}\cdot pid_{i}^{1}\right)$. Nevertheless, $\zeta_{i}$ is saved in the TPD, $s_{TA}$ is a random value, and therefore the attacker does not have the ability to obtain $TID_{SVP_{i}}$, since the hardness of the problem is related to the hardness of the Diffie–Hellman problem. So, the proposed EPBC-CPPA can ensure identity privacy-preserving.
- Unlinkability:
  A random number $\zeta_{i}$∈$Z_{q}^{*}$ is used in the proposed scheme to compute $pid_{i}=\left\{pid_{i}^{1},pid_{i}^{2}\right\}$ = $\left\{\zeta_{i}P,TID_{SVP_{i}}⊕h_{1}\left(\zeta_{i}P_{TA}\right)\right\}$. The vehicle periodically requests an update of its pseudonym IDs with timestamps svt that are only valid for brief periods. This scheme provides a list of them, to support unlinkability. Thus, no attacker could relate two or more signatures sent by the same vehicle for a long trip. Therefore, the proposed EPBC-CPPA scheme can fulfill the unlinkability requirement.
- Traceability and revocation:
  In the proposed SE-CPPA scheme, the TA has the ability to obtain the vehicle’s true identity from the received pseudonym ID that includes two parts—$pid_{i}^{1}=\zeta_{i}P$ and $pid_{i}^{2}=TID_{SVP_{i}}⊕h_{1}\left(\zeta_{i}P_{TA}\right)$. The TA uses its master private key $s_{TA}$, and calculates
  (10) $$\begin{array}{cc} \; & TID_{SVP_{i}}=pid_{i}^{2}⊕h_{1}\left(\zeta_{i}P_{TA}\right)\\ \; & =pid_{i}^{2}⊕h_{1}\left(\zeta_{i}s_{TA}\cdot P\right)\\ \; & =pid_{i}^{2}⊕h_{1}\left(s_{TA}pid_{i}^{1}\right) \end{array}$$
  After the vehicle’s true identity has been traced, the TA should revoke it on the database registration list, saving it in the CRL. Therefore, the proposed EPBC-CPPA scheme can fulfill traceability and revocation requirements.
- Resistance to replay attacks:
  The message of a signature tuple {$pid_{i}^{1}$, $pid_{i}^{2}$, $m_{i}$, svt, ts, $\delta_{m_{i}}$} in the proposed SE-CPPA scheme includes the current timestamp ts to generate the signature of the message $\delta_{m_{i}}=SK_{svt}\cdot h_{3}(m_{i}\left||ts\right)$, where $SK_{svt}=s_{TA}\cdot h_{3}(pid_{i}^{1}||pid_{i}^{2}\left||svt\right)$ and svt is only valid for a brief period of time. Hence, the proposed SE-CPPA scheme for VANETs can resist replay attacks.
- Resistance to modification attacks:
  Consistent with Theorem 2, we show that any alteration of the message of a signature tuple {$pid_{i}^{1}$, $pid_{i}^{2}$, $m_{i}$, svt, ts, $\delta_{m_{i}}$} can be determined by testing whether the equation *e*:$\left(\delta_{m_{i}}P\right)$ = *e*:$(h_{2}(pid_{i}^{1}$ $||pid_{i}^{2}\left||svt\right)$ $h_{3}(m_{i}||ts),P_{TA})$ holds or not. Hence, the proposed SE-CPPA scheme for VANETs can resist the modification attack.
- Resistance to impersonation attacks:
  Many researchers have resorted to storing the vehicle’s true identity in the TPD of the OBU to avoid its being compromised by an adversary. Nonetheless, a misbehaving vehicle could easily obtain the vehicle’s true identity saved in the TPD by launching a side-channel attack. To address this attack, the proposed SE-CPPA scheme frequently updates the ($TID_{SVP_{i}}$) in the TPD during SVP, where $TID_{SVP_{i}}=h_{1}(ID_{vi}\left||SVP\right)$ and SVP is a short period of validity. It has been stated that the vehicle’s true identity is used repeatedly; thus, if the $TID_{SVP_{i}}$ is not regularly updated, this will offer a wide opportunity for an attacker for impersonating and exploiting the registered vehicle’s true identity related to the safety messages. However, $TID_{SVP_{i}}$ is already updated before the vehicle can be impersonated and exploited by a misbehaving vehicle.
- Resistance to man-in-the-middle attacks:
  This SE-CPPA scheme executes mutual authentication between the signer and the recipient. If an attacker launches this attack, the attacker wants to send false messages for sharing with the the signer and the recipient. Nevertheless, based on Theorem 2, the attacker cannot succeed with this attack. Hence, the proposed SE-CPPA scheme for VANETs can resist man-in-the-middle attacks.

### 5.3. Security and Privacy Comparison

This subsection presents a comparison in terms of security and privacy requirements of the proposed SE-CPPA scheme with the existing schemes. Table 2 presents the results of this comparison. As presented in Table 2, all the existing schemes suffer from impersonation attacks by lunching side channel attacks to retrieve the vehicle’s true identity that saved on the OBU of the registered vehicle for broadcasting fake messages. In contrast, the proposed SE-CPPA scheme regularly updates the vehicle’s true identity at short intervals of time. Therefore, the impersonation attack is resisting by the proposed SE-CPPA scheme.

Furthermore, we know that the schemes proposed by Bayat et al. [36], Lei Zhang et al. [37], Bayat et al. [38], Pournaghi et al. [39] and Bayat et al. [41] for VANETs cannot satisfy all of the security analysis-based privacy requirements, as presented in Table 2. Nevertheless, the SE-CPPA scheme can satisfy all of the security analysis-based privacy requirements.

## 6. Performance Evaluation and Comparison

**Figure 4:**
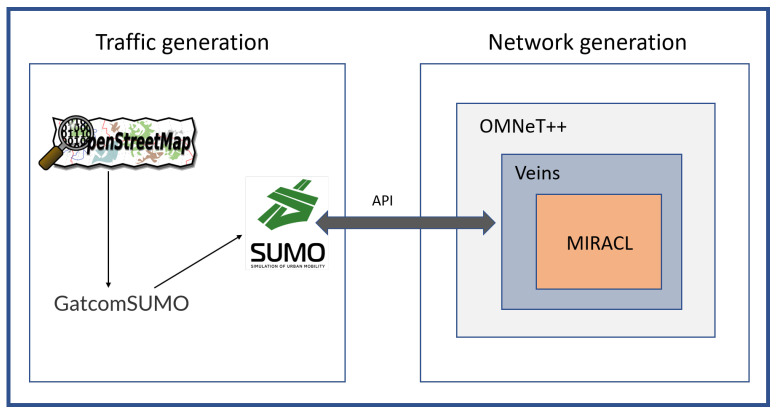
VANET simulation.

In this section, the performance evaluation of the proposed SE-CPPA scheme is analyzed in terms of computation and communication costs. Besides, the performance of the proposed SE-CPPA scheme is compared with Bayat et al. [36], Lei Zhang et al. [37], Bayat et al. [38], Pournaghi et al. [39], and Bayat et al. [41] through a simulation experiment. As shown in Figure 4, this paper uses OMNeT++ [46], Veins [47], MIRACL [48,49], OpenStreetMap [50], GatcomSUMO [51] and SUMO [52] to carry out simulation experiments for VANETs. OMNeT++ is a modular, component-based C++ simulation library for communication networks. Veins is combined with road traffic generation and network generation. MIRACL is a cryptography library used to execute cryptography operations for algorithms. OpenStreetMap is the most prominent crowd-sourced web-based mapping platform. GatcomSUMO is a graphical application used to simplify the utilization of VANET simulation, specifically the SUMO traffic and the OMNeT++ network generation. SUMO is a highly portable, multi-model traffic simulation. Table 3 presents the simulation experiment parameters.

### 6.1. Computation Cost and Comparison

The bilinear pairing is constructed on the 80 bits security level: *e*: $G_{1}$ ∗ $G_{1}$ → $G_{2}$, where $G_{1}$ is an additive group created on a super-singular EC *E*: $y^{2}=x^{3}+xmodp$ with embedding degree 2. For performance evaluation, the following bilinear pairing operations are considered.

- $T_{bp}$: The running time of the operation involving the bilinear pairing $\overset{-}{e}$ (P, Q), where $\overset{-}{P}$, $\overset{-}{Q}$∈$G_{1}$.
- $T_{bp\cdot pm}$: The running time of the operation of scalar multiplication $s\cdot \overset{-}{P}$ involved in the bilinear pairing, where $s\in Z_{q}^{*}$ and $\overset{-}{P}\in G_{1}$.
- $T_{bp\cdot pa}$: The running time of the operation of point addition $\overset{-}{P}$ + $\overset{-}{Q}$ involved in the bilinear pairing, where $\overset{-}{Q},\overset{-}{P}\in G_{1}$.
- $T_{M\cdot T\cdot P}$: The running time of the MapToPoint hash function.
- $T_{h}$: The running time of the secure cryptographic hash function.

Table 4 tabulates the single cryptographic operation time are taken into account. Table 5 presents a comparison of the computational costs of the proposed SE-CPPA and the other existing schemes. For simplicity, MSP denotes the message-signing phase, ISVP denotes the single-signature verification phase, BSVP denotes the batch-signature verification phase. These steps will be separately explained in the following,

#### 6.1.1. MSP

**Figure 5:**
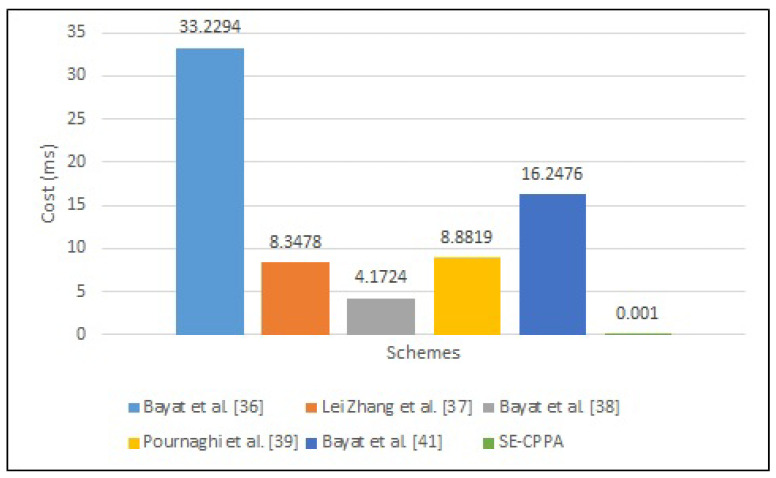
The comparison of message signing process.

The process of message signing in Bayat et al. [36] scheme consists of five bilinear pair operations $5T_{bp}$, a MapToPoint hash function operation $1T_{M\cdot T\cdot P}$ and two cryptographic hash function operations $2T_{h}$, hence, the whole computation cost of the message signing process is $5T_{bp}+1T_{M\cdot T\cdot P}+2T_{h}$. The process of message signing in Lei Zhang et al. [37] scheme consists of two MapToPoint hash function operations $T_{M\cdot T\cdot P}$ and three cryptographic hash function operations $3T_{h}$; hence, the whole computation cost of the message signing process is $2T_{M\cdot T\cdot P}+3T_{h}$. The process of message signing in Lei Zhang et al. [37] scheme consists of two MapToPoint hash function operations $2T_{M\cdot T\cdot P}$ and three cryptographic hash function operations $3T_{h}$; hence, the whole computation cost of the message signing process is $2T_{M\cdot T\cdot P}+3T_{h}$. The process of message signing in Bayat et al. [38] scheme consists of only one MapToPoint hash function operation $1T_{M\cdot T\cdot P}$; hence, the whole computation cost of the message signing process is $1T_{M\cdot T\cdot P}$. The process of message signing in the Pournaghi et al. [39] scheme consists of three scalar multiplication operations $3T_{bp\cdot pm}$, an addition point operation $1T_{bp\cdot pa}$, one MapToPoint hash function operation $1T_{M\cdot T\cdot P}$ and two cryptographic hash function operations $2T_{h}$; hence, the whole computation cost of the message signing process is $3T_{bp\cdot pm}+T_{bp\cdot pa}+1T_{M\cdot T\cdot P}+2T_{h}$. The process of message signing in Bayat et al. [41] scheme consists of two bilinear pair operations $2T_{bp}$, four scalar multiplication operations $4T_{bp\cdot pm}$, an addition point operation $1T_{bp\cdot pa}$, one MapToPoint hash function operation $1T_{M\cdot T\cdot P}$ and three cryptographic hash function operations $3T_{h}$; hence, the whole computation cost of the message signing process is $2T_{bp}+4T_{bp\cdot pm}+1T_{bp\cdot pa}+1T_{M\cdot T\cdot P}+3T_{h}$. The process of message signing in the proposed SE-CPPA scheme consists of only one cryptographic hash function operation $1T_{h}$, hence, the whole computation cost of the message signing process is $1T_{h}$. Figure 5 shows the comparison of message signing process.

#### 6.1.2. ISVP

**Figure 6:**
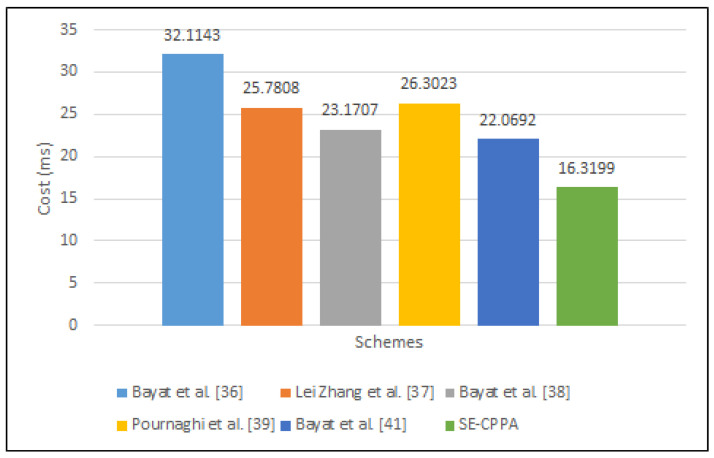
The comparison of single-signature verification process.

The process of single-signature verification in Bayat et al. [36] scheme consists of four bilinear pair operations $4T_{bp}$, three scalar multiplication operations $3T_{bp\cdot pm}$, a MapToPoint hash function operation $1T_{M\cdot T\cdot P}$ and two cryptographic hash function operations $2T_{h}$; hence, the whole computation cost of the single-signature verification process is $4T_{bp}+3T_{bp\cdot pm}+T_{M\cdot T\cdot P}+2T_{h}$. The process of single-signature verification in Lei Zhang et al. [37] scheme consists of three bilinear pair operations $3T_{bp}$, two MapToPoint hash function operations $1T_{M\cdot T\cdot P}$ and three cryptographic hash function operations $3T_{h}$; hence, the whole computation cost of the single-signature verification process is $3T_{bp}+2T_{M\cdot T\cdot P}+3T_{h}$. The process of single-signature verification in Bayat et al. [38] scheme consists of three bilinear pair operations $3T_{bp}$, a scalar multiplication operation $1T_{bp\cdot pm}$, and a MapToPoint hash function operation $1T_{M\cdot T\cdot P}$; hence, the whole computation cost of the single-signature verification process is $3T_{bp}+1T_{bp\cdot pm}+1T_{M\cdot T\cdot P}$. The process of single-signature verification in Pournaghi et al. [39] scheme consists of three bilinear pair operations $3T_{bp}$, three scalar multiplication operations $3T_{bp\cdot pm}$, a MapToPoint hash function operation $1T_{M\cdot T\cdot P}$ and a cryptographic hash function operation $1T_{h}$; hence, the whole computation cost of the single-signature verification process is $3T_{bp}+3T_{bp\cdot pm}+1T_{M\cdot T\cdot P}+1T_{h}$. The process of single-signature verification in the Bayat et al. [41] scheme consists of a bilinear pair operation $1T_{bp}$, four scalar multiplication operations $4T_{bp\cdot pm}$, an addition point operation $1T_{bp\cdot pa}$, a MapToPoint hash function operation $1T_{M\cdot T\cdot P}$, and two cryptographic hash function operations $2T_{h}$; hence, the whole computation cost of the single-signature verification process is $1T_{bp}+4T_{bp\cdot pm}+1T_{bp\cdot pa}+1T_{M\cdot T\cdot P}+2T_{h}$. The process of single-signature verification in the proposed SE-CPPA scheme consists of two bilinear pair operations $2T_{bp}$, two scalar multiplication operations $2T_{bp\cdot pm}$, and two cryptographic hash function operations $2T_{h}$; hence, the whole computation cost of the single-signature verification process is $2T_{bp}+2T_{bp\cdot pm}+2T_{h}$. Figure 6 shows the comparison of single-signature verification process.

#### 6.1.3. BSVP

**Figure 7:**
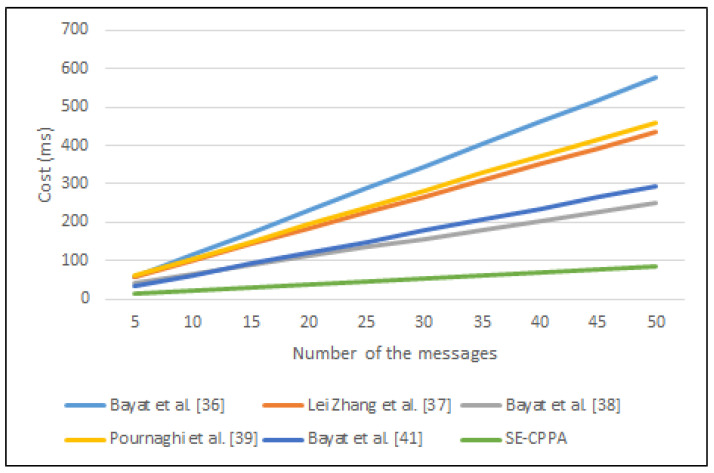
The comparison of batch-signature verification process.

The process of batch-signature verification in Bayat et al. [36] scheme consists of n bilinear pair operations $nT_{bp}$, *n* scalar multiplication operations $nT_{bp\cdot pm}$, *n* MapToPoint hash function operations $nT_{M\cdot T\cdot P}$ and n cryptographic hash function operations $nT_{h}$, hence, the whole computation cost of the batch-signature verification process is $nT_{bp}+nT_{bp\cdot pm}+nT_{M\cdot T\cdot P}+nT_{h}$. The process of batch-signature verification in Lei Zhang et al. [37] scheme consists of 3 bilinear pair operations $3T_{bp}$, 2*n* MapToPoint hash function operations $2nT_{M\cdot T\cdot P}$ and 3*n* cryptographic hash function operations $3nT_{h}$, hence, the whole computation cost of the batch-signature verification process is $3T_{bp}+\left(2n\right)T_{M\cdot T\cdot P}+\left(3n\right)T_{h}$. The process of batch-signature verification in Lei Zhang et al. [37] scheme consists of 3 bilinear pair operations $3T_{bp}$, 2*n* MapToPoint hash function operations $2nT_{M\cdot T\cdot P}$ and 3*n* cryptographic hash function operations $3nT_{h}$, hence, the whole computation cost of the batch-signature verification process is $3T_{bp}+\left(2n\right)T_{M\cdot T\cdot P}+\left(3n\right)T_{h}$. The process of batch-signature verification in Bayat et al. [38] scheme consists of 3 bilinear pair operations $3T_{bp}$, *n* scalar multiplication operations $nT_{bp\cdot pm}$ and *n* MapToPoint hash function operations $nT_{M\cdot T\cdot P}$, hence, the whole computation cost of the batch-signature verification process is $3T_{bp}+nT_{bp\cdot pm}+nT_{M\cdot T\cdot P}$. The process of batch-signature verification in Pournaghi et al. [39] scheme consists of 3 bilinear pair operations $3T_{bp}$, 3*n* scalar multiplication operations $3nT_{bp\cdot pm}$, *n* MapToPoint hash function operations $nT_{M\cdot T\cdot P}$ and *n* cryptographic hash function operations $nT_{h}$, hence, the whole computation cost of the batch-signature verification process is $3T_{bp}+\left(3n\right)T_{bp\cdot pm}+nT_{M\cdot T\cdot P}+nT_{h}$. The process of batch-signature verification in Bayat et al. [41] scheme consists of (4+n) scalar multiplication operations $\left(4+n\right)T_{bp\cdot pm}$, n addition point operations $nT_{bp\cdot pa}$, *n* MapToPoint hash function operations $nT_{M\cdot T\cdot P}$ and *n* cryptographic hash function operations $nT_{h}$, hence, the whole computation cost of the batch-signature verification process is $\left(4+n\right)T_{bp\cdot pm}+nT_{M\cdot T\cdot P}+\left(n\right)T_{bp\cdot pa}+nT_{h}$. The process of batch-signature verification in the proposed SE-CPPA scheme consists of a bilinear pair operations $T_{bp}$, *n* scalar multiplication operations $nT_{bp\cdot pm}$ and 2*n* cryptographic hash function operations $2nT_{h}$, hence, the whole computation cost of the batch-signature verification process is $T_{bp}+nT_{bp\cdot pm}+\left(2n\right)T_{h}$. Figure 7 shows the comparison of batch-signature verification process.

### 6.2. Communication Overhead and Comparison

This section analyses and compares the communication cost of the proposed SE-CPPA and other schemes. The main focus is the communication cost involved in the pseudonym-IDs, signatures, and timestamps for the signature tuple. Table 6 presents the costs of several bilinear pairing operations.

The size of the signature tuple {$ID_{i}$, $M_{i}$, $\sigma_{i}$, $T_{i}$ } in the scheme of Bayat et al. [36] is 128 × 3 + 4 × 1 = 388 bytes, which contains three elements in $G_{1}$ ($ID_{i1},ID_{i2},\sigma_{i}\in G_{1}$) and one timestamp ($T_{i}$), where $ID_{i}=\left\{ID_{i1},ID_{i2}\right\}$. The size of the signature tuple {$m_{i}$, $PPID_{i,t}$, $\sigma_{i}$ } in the scheme of Lei Zhang et al. [37] is 128 × 2 = 256 bytes, which contains two elements in $G_{1}$ ($PPID_{i,t}$, $\sigma_{i}\in G_{1}$). The size of the signature tuple {$M_{i}$, $pid_{i}$, $\sigma_{i}$ } in the scheme of Bayat et al. [38] is 128 × 2 + 20 = 276 bytes, which contains two elements in $G_{1}$ ($ID_{i1},\sigma_{i}\in G_{1}$), one outputs regarding the hash function ($ID_{i2}\in Z_{q}^{*}$) and one timestamp ($T_{i}$), where $pid_{i}$= $PID_{1},PID_{2}$. The size of the signature tuple {$pID_{i}$, $\sigma_{i}$, $M_{i}$, $ID_{RSU}$ } in the scheme of Pournaghi et al. [39] is 128 × 3 + 20 = 404 bytes, which contains three elements in $G_{1}$ ($ID_{i1},ID_{i2},\sigma_{i}\in G_{1}$) and one timestamp ($T_{i}$), where $ID_{i}=\left\{ID_{i1},ID_{i2}\right\}$. The size of of the signature tuple {$V,m,r,T_{i1},$ $T_{i2},T_{i3},PID_{i},ts$} in Bayat et al. [41] is 128 × 4 + 20 × 2 + 4 × 2 = 556 bytes, which contains four elements in $G_{1}$ ($T_{i1},$ $T_{i2},T_{i3},PID_{i}\in G_{1}$), two outputs regarding the hash function ($V,r\in Z_{q}^{*}$) and one timestamp (ts). The size of of the signature tuple {$pid_{i}^{1}$, $pid_{i}^{2}$, $m_{i}$, svt, ts, $\delta_{m_{i}}$} in the proposed SE-CPPA scheme is 128 × 1 + 20 × 2 + 4 × 2 = 216 bytes, which contains one element in $G_{1}$ ($pid_{i}^{1}\in G_{1}$), two outputs regarding the hash function ($pid_{i}^{2},\delta_{m_{i}}$ $\in Z_{q}^{*}$) and two timestamps (svt,ts).

**Figure 8:**
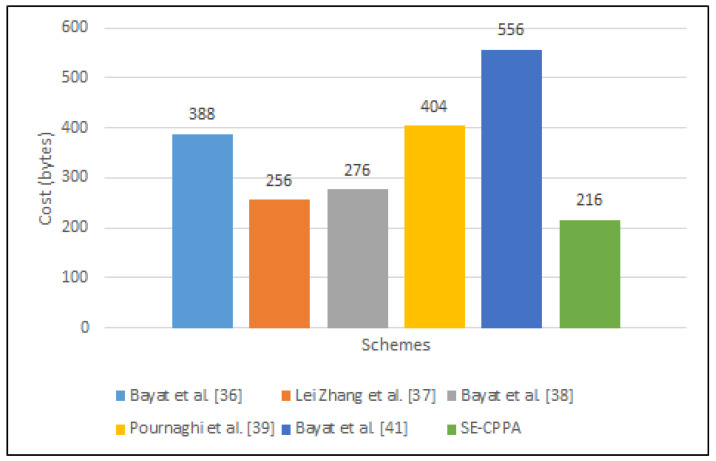
Communication overhead comparison based on bilinear pair.

The communication cost of each scheme is presented in Table 7. Figure 8 compares the communication overheads of the SE-CPPA and the other schemes.

## 7. Conclusions

In this paper, a Secure and Efficient Conditional Privacy-Preserving Authentication (SE-CPPA) scheme for VANETs has been proposed. In contrast with the existing schemes, it has the ability to resist impersonation attacks, since it frequently updates the vehicle’s true identity stored on a TPD on the vehicle. In a region with dense traffic, the batch-signature verification process in the SE-CPPA scheme efficiently checks a large number of the signature tuple messages sent from different components in the VANET. The security proof showed that the proposed SE-CPPA scheme resists security attacks and fulfills requirements regarding security and privacy. Lastly, due to the fact that the proposed SE-CPPA scheme does not employ time-consuming operations involving the MapToPoint hash function while signing and verifying the messages, it has lower overhead costs in contrast to the existing schemes. Hence, SE-CPPA has a more efficient performance regarding computational and communication costs. In the future work, further performances in terms of end-to-end delay and throughput will be briefly analyzed and introduced by using OMNeT++ and SUMO simulations.

## Figures and Tables

**Table 1:** Notation and their description.

Notation	Description
TA	The Trusted Authority
OBU	The On-Board Unit
RSU	The Road-Side Unit
TPD	The Tamper Proof Device
CRL	Certificate Revocation List
*P*	The base generator P ∈ $G_{1}$
$h_{1},h_{2},h_{3}$	Three secure hash functions
$ID_{vi}$, Pwd	Identity and password of vehicle
$TID_{SVP_{i}}$	Vehicle’s true identity
SVP, svp	Short valid period of vehicle’s signature key
svt	Short valid period of vehicle’s true identity
$\delta_{m_{i}}$, $\delta_{RJ}$	The message signature
$\zeta_{i}$, *k*	Random integer
$s_{TA},P_{TA}$	The private/public keys of TA
$SK_{svt}$	The signature key of vehicle
⊕	XOR operator
$\gamma_{i}$	a random vector
$m_{i}$	Safety traffic-related messages
‖	Concatenation operation
ts	Current timestamp

**Table 2:** Security analysis-based privacy requirements.

Requirements	Bayat et al. [36]	Lei Zhang et al. [37]	Bayat et al. [38]	Pournaghi et al. [39]	Bayat et al. [41]	SE-CPPA
Message Integrity and Authentication	✓	✓	✓	✓	✓	✓
Identity Privacy-Preserving	✓	✓	✓	✓	✓	✓
Unlinkability	✓	✓	✓	✓	✗	✓
Traceability and Revocation	✗	✗	✓	✓	✓	✓
Resistance to Modification Attacks	✓	✓	✓	✓	✓	✓
Resistance to Replay Attacks	✓	✓	✗	✗	✓	✓
Resistance to Man-in-the-Middle Attacks	✓	✓	✓	✓	✓	✓
Resistance to Impersonation Attacks	✗	✗	✗	✗	✗	✓

**Table 3:** Simulation experiment parameters.

Parameters	Value
Simulation time	200 s
Playground size	*x* = 3463 m, *y* = 4270 m and *z* = 50 m
Mac Layer	IEEE 1609.4
Physical Layer	IEEE 802.11 p
Maximum transmission	20 mW
Bit rate	6 Mbps

**Table 4:** The single cryptographic operation time.

Cryptography Operations	Time (ms)
$T_{bp}$	5.811
$T_{bp\cdot pm}$	1.5654
$T_{bp\cdot pa}$	0.0106
$T_{M\cdot T\cdot P}$	4.1724
$T_{h}$	0.001

**Table 5:** Cost of computation comparison.

Schemes	MSP	ISVP	BSVP
Bayat et al. [36]	$5T_{bp}+T_{M\cdot T\cdot P}+2T_{h}$	$4T_{bp}+3T_{bp\cdot pm}+T_{M\cdot T\cdot P}+2T_{h}$	$nT_{bp}+nT_{bp\cdot pm}+nT_{M\cdot T\cdot P}+nT_{h}$
Lei Zhang et al. [37]	$2T_{M\cdot T\cdot P}+3T_{h}$	$3T_{bp}+2T_{M\cdot T\cdot P}+3T_{h}$	$3T_{bp}+\left(2n\right)T_{M\cdot T\cdot P}+\left(3n\right)T_{h}$
Bayat et al. [38]	$1T_{M\cdot T\cdot P}$	$3T_{bp}+1T_{bp\cdot pm}+1T_{M\cdot T\cdot P}$	$3T_{bp}+nT_{bp\cdot pm}+nT_{M\cdot T\cdot P}$
Pournaghi et al. [39]	$3T_{bp\cdot pm}+T_{bp\cdot pa}+1T_{M\cdot T\cdot P}+2T_{h}$	$3T_{bp}+3T_{bp\cdot pm}+1T_{M\cdot T\cdot P}+1T_{h}$	$3T_{bp}+\left(3n\right)T_{bp\cdot pm}+nT_{M\cdot T\cdot P}+nT_{h}$
Bayat et al. [41]	$1T_{bp}+4T_{bp\cdot pm}+1T_{M\cdot T\cdot P}+1T_{bp\cdot pa}+3T_{h}$	$2T_{bp}+4T_{bp\cdot pm}+1T_{M\cdot T\cdot P}+1T_{bp\cdot pa}+3T_{h}$	$\left(4+n\right)T_{bp\cdot pm}+nT_{M\cdot T\cdot P}+\left(n\right)T_{bp\cdot pa}+nT_{h}$
SE-CPPA	$1T_{h}$	$2T_{bp}+2T_{bp\cdot pm}+2T_{h}$	$T_{bp}+nT_{bp\cdot pm}+\left(2n\right)T_{h}$

**Table 6:** The costs of several bilinear pairing operations.

Items Size	Cost (Bytes)
$\overset{-}{P}$	64
The elements in $G_{1}$	128
The output of a hash function	20
The output of timestamp	4

**Table 7:** Communication cost comparison.

Schemes	Broadcasting One Message	Broadcasting *n* Messages
Bayat et al. [36]	388	388*n*
Lei Zhang et al. [37]	256	256*n*
Bayat et al. [38]	276	276*n*
Pournaghi et al. [39]	404	404*n*
Bayat et al. [41]	556	556*n*
SE-CPPA	216	216*n*

## Data Availability

Data sharing not applicable.
